# Outbreaks of healthcare-associated infections linked to water-containing hospital equipment: a literature review

**DOI:** 10.1186/s13756-021-00935-6

**Published:** 2021-05-10

**Authors:** Wing-Kee Yiek, Olga Coenen, Mayke Nillesen, Jakko van Ingen, Edmée Bowles, Alma Tostmann

**Affiliations:** Department of Medical Microbiology, Radboud Centre for Infectious Diseases, Radboud University Medical Centre, Nijmegen, The Netherlands

**Keywords:** Healthcare-associated infection, Nosocomial infection, Infection prevention and control, Outbreak, Hospital management

## Abstract

**Background:**

Healthcare-associated infections (HAIs) are a significant cause of morbidity and mortality in hospitalized patients. Water in the environment can be a source of infection linked to outbreaks and environmental transmission in hospitals. Water safety in hospitals remains a challenge. This article has summarized available scientific literature to obtain an overview of outbreaks linked to water-containing hospital equipment and strategies to prevent such outbreaks.

**Methods:**

We made a list of water-containing hospital equipment and devices in which water is being used in a semi-closed circuit. A literature search was performed in PubMed with a search strategy containing the names of these medical devices and one or more of the following words: outbreak, environmental contamination, transmission, infection. For each medical device, we summarized the following information: the function of the medical device, causes of contamination, the described outbreaks and possible prevention strategies.

**Results:**

The following water-containing medical equipment  or devices were identified: heater-cooler units, hemodialysis equipment, neonatal incubators, dental unit waterlines, fluid warmers, nebulizers, water traps, water baths, blanketrol, scalp cooling, and thermic stimulators. Of the latter three, no literature could be found. Of all other devices, one or more outbreaks associated with these devices were reported in the literature.

**Conclusions:**

The water reservoirs in water-containing medical devices can be a source of microbial growth and transmissions to patients, despite the semi-closed water circuit. Proper handling and proper cleaning and disinfection can help to reduce the microbial burden and, consequently, transmission to patients. However, these devices are often difficult to clean and disinfect because they cannot be adequately opened or disassembled, and the manufacturer’s cleaning guidelines are often not feasible to execute. The development of equipment without water or fluid containers should be stimulated. Precise cleaning and disinfection guidelines and instructions are essential for instructing healthcare workers and hospital cleaning staff to prevent potential transmission to patients.

## Introduction

Healthcare-associated infections (HAIs) are an important cause of morbidity and mortality in hospitalized patients. HAIs are defined as infections occurring during or after the process of care that were not present or incubating at the time of the patient’s admission to a hospital or other healthcare facility [1].

Environmental contamination plays a role in the transmission of microorganisms that can cause infections. Water should be considered an important source of infection due to the numerous occasions of exposure [1, 2]; these include the complex hospital water systems as well as water-containing tools and machinery used in hospital facilities. Because of the greater susceptibility of patients in hospitals and/or long-term care or rehabilitation centers to infections, waterborne pathogens are more likely to cause infection in healthcare institutions than in the healthy population [3].

Water serves many function in a healthcare environment and it is estimated that 65% of HAIs are associated with wet biofilms, or the presence of moisture or liquid [4]. Transmission of pathogens from a water reservoir may occur by direct and indirect contact, ingestion and aspiration of contaminated water, or inhalation of aerosols [2].

A sizable proportion of HAIs can be prevented by proper handling of medical devices, high levels of hand hygiene compliance, environmental hygiene, use of personal protective equipment, and screening and isolation [4]. Cleaning is used to reduce the microbial growth, but methods can vary between hospitals [4]. However, cleaning and disinfection of water containing devices is often not possible because not every surface of the device can be reached.

Water safety in hospitals remains a challenge [5]. There are many potential reservoirs that could potentially host pathogens and cause outbreaks. Even for devices with a semi-closed or a closed circuit where a patient is not in direct contact with the water, it is possible that the patient gets exposed, such as through aerosols or water splashes from the tubing systems or the reservoir access. This can happen when the circuit is manually opened to change the water and/or the tubing or when there is a ventilation circuit. The exact number of outbreaks and transmission from water containing medical equipment is unknown [3].

In this review, we summarize available scientific literature to obtain an overview of outbreaks linked to water-containing hospital equipment and strategies to prevent such outbreaks.

## Methods

### Search strategy

We started with compiling a list of water containing medical equipment and devices that are being used in hospitals, including our own hospital. The criteria for the equipment were that they had a water reservoir, creating a semi-closed or a closed circuit. The patient and patient environment would not be in direct contact with the water. To complement the list of medical equipment, we performed a literature search, using PubMed. The complete search strategy can be found in Table 1.

**Table 1:** The following search terms were used to identify different medical equipment

	Hospital* OR healthcare OR hospital units OR hospital environment OR dental facilities OR intensive care OR healthcare environment
AND	Healthcare-acquired infection OR infection, nosocomial OR hospital-acquired OR healthcare-associated infection* OR hospital-associated infection* OR outbreak OR hospital outbreak* OR healthcare outbreak* OR waterborne outbreak OR waterborne pathogen OR waterborne diseases OR cross infection OR contamination OR colonization OR infection prevention OR infection control OR biofilm OR air sampling OR transmission
AND	hospital water OR water microbiology OR water reservoir OR water system OR disease reservoir

The keywords that are highlighted indicate that these are Medical Subject Headings (MeSH) Terms

Only literature that was available in English or Dutch were included from the time period of January 1980 till December 2019. Additionally, articles that were unavailable in PubMed and articles that did not include human infections were excluded.

Based on the previously mentioned search strategy and the compiled list, the following medical equipment were distinguished. The following inventory with key words was made for each medical device (see Table 2). These key words were combined with previously mentioned search strategy to collect the number of articles and its outbreaks.

**Table 2:** An inventory list of all water containing medical equipment and their corresponding search strings

Water containing medical equipment	Key words	Number of hits on PubMed
Heater-cooler unit	(Heater-cooler OR heater-cooler units OR heater-cooler devices) AND (mycobacterium OR chimaera) Previous search string was not combined with the key words for the heater-cooler unit	72
Fluid warmer	Fluid warmer OR blood warmer OR warming device AND hypothermia	0
Blanketrol	Hypothermia AND Blanket OR blanketrol OR roll OR heat roll	0
Reverse Osmosis/hemodialysis	Reverse osmosis OR hemodialysis OR hemodialysis OR dialysis water OR dialysis OR dialysate OR dialysis fluid	24
Scalp cooling	Chemotherapy OR chemo AND scalp cooling OR cold caps OR cold membrane	0
Incubators	NICU OR neonate OR neonatal unit OR incubator	56
Nebulizers	Nebulizers and vaporizers OR nebulizer*	1
Water traps	Water trap OR ventilator trap OR ventilation circuit OR ventilator OR condensate OR humidifier trap	13
Dental units	Dental unit OR dental stand-alone OR dental water OR dental unit waterline	45
Thermic stimulator	Neuro sensory OR sensory analyser OR sensory analyser or thermic stimulator	0
Water baths	Water baths OR water-baths OR thawing baths AND transfusion OR cryoprecipitate OR thawing OR frozen	1

The number of hits per medical device is not representative of the actual number of outbreaks related to that particular device

References of articles and systematic reviews provided further literature and links to reported outbreaks. Grey literature such as protocols or reports of public health websites (CDC, WHO, FDA, ECDC etc.) were consulted as well. Studies identified through a handsearching process were also included.

### Inclusion and exclusion criteria

Literature was only included if the outbreak or transmission was caused by a contaminated water reservoir of a medical equipment itself. Contamination through healthcare workers who came in contact with certain water reservoir was also taken into account.

Articles were excluded when the outbreak was caused by improper cleaning or disinfection of the equipment or when the outbreak was caused by using contaminated resources, such as contaminated disinfectants or contaminated medication.

Articles regarding tap or potable water were excluded, as well as literature regarding non-medical devices, such as room humidifiers. For each medical equipment the following was described if available: the function of the medical device, the cause of contamination, the described outbreaks and possible prevention strategies.

## Results

The first search string to compile the list of medical equipment resulted in 629 articles. Literature on outbreaks associated with the water containing medical equipment was found for 8 of the 11 different devices on our list. The number of articles per device can be found in Table 6. No literature on outbreaks or associated transmission could be found regarding the blanketrol, the scalp cooling used for chemotherapy and thermic stimulators.

### Heater-cooler units

**Fig. 1:**
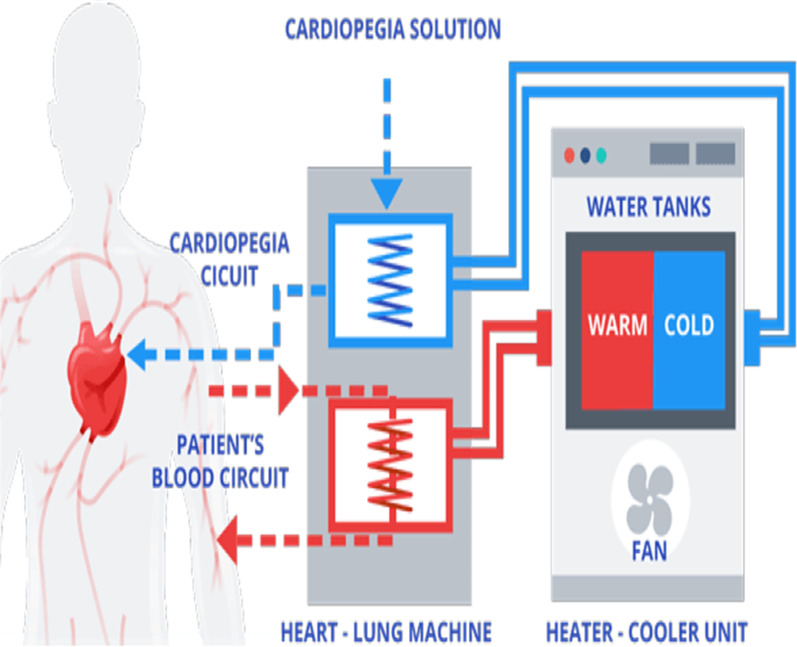
An example of a heater-cooler unit [8] (The blanket circuit is not included in this figure)

Heater-cooler units (HCUs) are stand-alone devices connected to a cardiopulmonary bypass machine and are used to regulate the temperature of the blood by using water as a heat exchanger (see Fig. 1). They are often used for open-chest heart surgeries on extracorporeal circulation and is usually situated inside the operating room. For patients receiving extracorporeal membrane oxygenation (ECMO) a HCU is also used on intensive care units. The water is stored in a water reservoir from where pumps supply the tubing of three circuits. The first one is a patient circuit to cool and warm the patient’s blood, the second one is a cardioplegia circuit to cool the cardioplegia solution and the third is a blanket circuit for additional external cooling and or warming of the patient. The water is not intended to have contact with the patient or their blood, but the circuit is not airtight. The cooling of HCU water is accomplished with a radiator with a fan. These fans produce a far-reaching airflow [6, 7].

Water reservoirs such as those from HCUs provide favourable environmental conditions for the growth of microorganisms such as non-tuberculous mycobacteria. Non-tuberculous mycobacteria, in particular  *Mycobacterium chimaera*, are opportunistic human pathogens and are intrinsically resistant to most disinfectants and most classes of antibiotics. Because the water systems of HCUs are not airtight and the cooling fans produce such an airflow, they can potentially expose patients to aerosols containing non-tuberculous mycobacteria during surgery [9].

All currently reported cases of *Mycobacterium chimaera* infection were linked to the *Stöckert 3T HCU* by *LivaNova* before September 2014. Cases have been reported in patients who have undergone surgery in Europe (The Netherlands, Germany, UK, France, Switzerland, Ireland and Spain) as well as in the US, Australia, Canada and Hong Kong [9, 10].

Patients undergoing cardiac surgery involving cardiopulmonary bypass where their body temperature are regulated by HCUs are at risk of exposure and infection. Patients with a surgery longer than two hours and patients who received an implantation of prosthetic material had a higher risk of infection [10].

### Infection control

Risk mitigation is quite challenging for HCU. Several studies have shown that *Mycobacterium chimaera* proved virtually ineradicable from HCUs once it has colonized the water circuit, despite more intensive disinfection strategies and approaches. This is due to the lipid-rich cell wall of the bacteria and the high concentration within biofilms, making them highly resistant to standard disinfectants and making them highly amenable to aerosolization from water circuits [11]. The disinfection protocol of 3 T HCUs has been revised multiple times and intensified. Only the 3 T HCU has been linked to the global *M. chimaera* outbreak. Other HCUs like the Maquet HCU30 showed contamination but without aerosolization and Maquet HCU40 tested negative for *Mycobacterium chimaera* [11, 14].

Based hereupon, the ECDC has advised EU member states to relocate HCUs outside of the operating room where feasible; if not feasible, place at maximal distance from the operating table with the exhaust vent directed away from the patient and close to the air suction exhaust. In addition strict adherence to cleaning and disinfection protocols or a change to another HCU brand or type is encouraged [12, 13]. The FDA also recommends to only use water that has been passed through a filter of less than or equal to 0.22 microns. Tap water, deionized and sterile water created through reverse osmosis are not recommended because they may promote corrosion of the metal components. However, unlike the ECDC, the FDA only recommends to direct and channel the exhaust away from the patient [13].

### Hemodialysis equipment

**Fig. 2:**
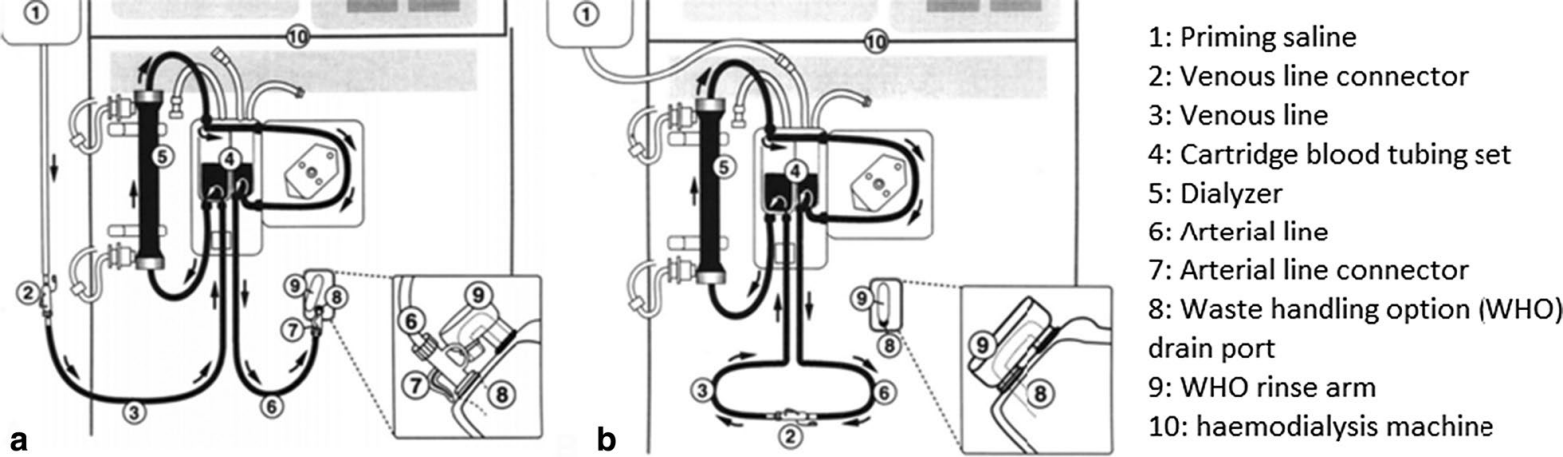
A diagram of components of a hemodialysis system, illustrating blood pathway. **a** Configuration of tubing for priming of blood pathway with sterile saline. **b** Configuration of tubing for recirculation of priming saline [20]

Patients who receive hemodialysis are at increased risk of bloodstream infections due to repeated vascular access. This is usually linked to inadequate catheter care, contamination of water supply, defects in membrane integrity or reprocessed dialysers [14–16]. The water for hemodialysis is called dialysate and is not required to be sterile, but to reduce the risk of bloodstream infection the number of bacteria present must be below a threshold [17]. Primary waterborne microbial contaminants of dialysis fluids are gram-negative bacteria and non-tuberculous mycobacteria. *Pseudomonas aeruginosa* and *Burkholderia cepacia* are frequently isolated. These bacteria can form biofilms that allow them to attach to surfaces, such as dialysate containers or feed hoses. Dialysis water treatment removes chemical and microbial contaminants. Water treatment includes reverse osmosis (RO), which is the primary water purification process of choice [18, 19] (Fig. 2).

We found 11 reports of hemodialysis-associated outbreaks. Most of them were linked to hemodialysis units, patients became infected because the dialysis water exceeded the maximum amount of chemical and microbial contaminants due to lack of disinfection. Inadequate disinfection allows concentrations of bacteria to propagate [18].

Additionally, treated water is often stored in reservoirs where it is distributed to dialysis machines. It has been observed that water stagnancy contributed to bacterial contamination of the water in the pipe systems [19].

Other sources of contamination were related to inadequate cleaning procedures that left leaking connections of the RO tubing. Biofilm-forming bacteria and other microorganisms present in cleaning solutions could have entered the water system through this opening. It is known that tubing connections are critical segments of the system and are a possible site for biofilm development [14].

### Infection control

In 50% of the known outbreaks associated to hemodialysis machines, the reuse of the dialyzers was the presumed cause. Reusing dialyzers is done for reducing the incidence of first use syndrome, which is hypersensitivity to ethylene oxide and for economic reasons. Reprocessing or reusing dialyzers makes them more vulnerable to contamination from water [18]. This should therefore be taken into account.

The most common breaches in infection control during hemodialysis include: errors in dialyzer processing, backflow into blood lines from WHO ports, cross-contamination with dialysis fluid, and undetected membrane leaks [18, 22]. WHO ports are designed to dispose the saline used to flush a dialyzer before the machine is used for a patient. Outbreaks caused by these ports were observed in three separate outbreaks (see Table 3) [21, 22]. It was also seen in one study that receiving dialysis via a central venous catheter (CVC) instead of an arterio-venous shunt was a risk factor, as this caused cross-contamination of CVCs from the WHO port. This was likely caused by the reflux from the waste drain line into the WHO, and bacterial growth in the nutrient-rich environment of the WHO [22]. In addition, WHO ports were able to enter blood line tubing by at least two routes. During priming they could ascend directly through the lumen of the connector into the arterial line tubing. The second route is the inadvertent inoculation of the open ends of blood line tubing by technicians during reconfiguration of the lines or attachment to the patient’s vascular access [20]. When technicians had to reconfigure the lines, it was also seen that the patient’s vascular access was temporarily being connected to a ‘dirty’ WHO priming connector, which had been sitting in an open WHO port where thus reflux may have occurred [22]. It was also often observed that technicians left the WHO priming connector inserted in the WHO port, which prevented the rinse arm from properly closing over the WHO port to allow flushing of the WHO. This results in using the same previously used WHO priming connector, rather than the new clean one [22].

**Table 3:** An overview of all the hemodialysis related outbreaks in hospitals with the number of cases with the respective strains

Study	Country	Strain	N of cultures, (%)	N of cases, (%)	Mortality, N (%)	Cause of the outbreak
CDC [21]	Canada	*Enterobacter cloacae*	NA	9	Unknown	Waste handling option (WHO) port^a^: Incompetent one-way valve of the drain line waste of the dialysis machine → backflow
CDC and Wang et al. [21, 22]	United States	1*. Enterobacter cloacae* 2. *Pseudomonas aeruginosa* 3. *Escherichia coli*	1. 14 out of 26 machines (53.8) 2. 7 out of 26 machines (26.9) 3. Unknown	N = 10 1. *6 (60.0)* 2. *4 (40.0)* 3. *2 (20.0)* Two were polymicrobial	Unknown	WHO port^a^: Incompetent valves on waste drain lines. Cross-contamination of hemodialysis central venous catheters from the WHO port: Reflux from the waste drain line into the WHO^a^ port Bacterial growth in the nutrient-rich environment of the WHO^a^ port
Rao et al. [23]	United States	*Phialemonium curvatum*	2 out of 19 treatment stations (10.5)	2	Unknown	Malfunction and improper maintenance of WHO^a^ port
CDC [21]	Israel	1. *Enterobacter cloacae* 2. *Pseudomonas aeruginosa* 3. *Escherichia coli* 4. *Stenotrophomonas maltophilia*	6 out of 13 dialysis machines (46.1)	N = 8 1. 2 (25.0) 2. 3 (37.5) 3. 4 (50.0) 4. 1 (12.5)	Unknown	Backflow of the WHO^a^ port
Yan et al. [24]	China	*Burkholderia cepacia*	NA	9	Unknown	Reverse osmosis water
Souva et al. [14]	Brazil	*Burkholderia cepacia*	33	28	Unknown	Inadequate cleaning procedures → leaking connections of the reverse osmosis tubing
Magalhaes et al. [16]	Brazil	1. *Burkholderia cepacia* 2. *Staphylococcus aureus* 3. *Streptococcus agalactiae* 4. *Enterobacter aerogenes* 5. *Pseudomonas aeruginosa* 6. *Acinetobacter baumanii*	37	N = 14 1. 6 (42.9) 2. 4 (28.6) 3. 1 (7.1) 4. 1 (7.1) 5. 1 (7.1) 6. 1 (7.1)	Unknown	Probable colonization in the reverse osmosis membrane
Nazemi et al. [25]	Iran	1. *Legionella pneumophila* 2. *Pseudomonas aeruginosa* 3. *Staphylococcus aureus* 4. *Escherichia coli* 5. *Burkholderia cepacia* 6. *Gram-positive cocci*	24 out of 50 samples (48.0) 1. 4 (16.7) 2. 6 (25.0) 3. 3 (12.6) 4. 3 (12.5) 5. 2 (8.3) 6. 6 (25.0)	NA	NA	Most commonly during reverse osmosis, in the storage tank and dialysate effluent
Kaitwatcharachai et al. [15]	Thailand	*Burkholderia cepacian*	NA	9	1 (11.1)	Deionized water used to dilute the dialysate concentrate and the in-use dialysis fluid
Oie et al. [26]	Japan	1. *Moraxella* spp 2. *Pseudomonas aeruginosa* 3. *Gram-negative bacteria* 4. Sp*hingomonas paucimobilis* 5. *Ralstonia pickettii* 6. *Pseudomonas stutzeri* 7. *Pasteurella multocida*	17 out of 40 (42.5) dialysate samples showed bacterial count exceeding the AAMI standard	1. 10 (25.0) 2. 8 (20.0) 3. 6 (15.0) 4. 5 (12.5) 5. 2 (5.0) 6. 2 (5.0) 7. 2 (5.0)	NA	Tubing within the dialysis machine may be the site of biofilm development
Arnow et al. [20]	United States	1. *Enterobacter cloacae* 2. *Stenotrophomonas maltophilia* 3. *Enterococcus faecalis* 4. *Acinetobacter baumannii* 5. *Candida parapsilosis* 6. *Candida tropicalis* 7. *Pseudomonas aeruginosa* 8. *Alcaligenes xylosoxidans* 9. *Serratia marcescens* 10. *Acinetobacter Iwoffi* 11. *Enterococcus faecium* 12. *Klebsiella pneumoniae* 13. *Flavobacterium* sp*ecies* 14. *Lactobacillus* sp*ecies* 15. *Burkholderia pickettii* 16. *Pseudomonas stutzeri*	Unknown	N = 29 1. 7 (24.1) 2. 7 (24.1) 3. 6 (20.7) 4. 5 (17.2) 5. 4 (13.8) 6. 3 (10.3) 7. 3 (10.3) 8. 3 (10.3) 9. 2 (6.9) 10. 1 (3.4) 11. 1 (3.4) 12. 1 (3.4) 12. 1 (3.4) 13. 1 (3.4) 14. 1 (3.4) 15. 1 (3.4)	1^b^ (3.4)	Microbial growth in the outer portion of the WHO^a^ port despite circulation of a disinfectant through the fluid pathway deeper in the lumen
Olver et al. [27]	United Kingdom	*Enterococcus faecali*	NA	2	NA	WHO^a^ port cannot be cleaned adequately

^a^WHO port: Waste handling option port: a drain port to dispose of saline used to flush the dialyser before patient use

^b^Unrelated to the bloodstream infection

Studies have also observed that an ultrafiltration membrane before the entrance of dialysate into the dialyzer is effective in preventing the microbial contamination of dialysate [26, 28]. Routine weekly disinfection that is recommended by the manufacturer is likely ineffective in removing any biofilm that could form, and it does not disinfect the outer rim of the WHO port or the tip of the WHO rinse arm. The dialysis lines, the WHO connector, the rim, and the tip are all in close proximity or even in contact with each other, that cross-contamination of patient dialysis lines may readily occur [22].

Other studies have shown that the use of ultrapure water, defined as microbial contamination of < 0.1 CFU/mL and endotoxin contamination of < 0.03 IU/mL, lead to a significant decrease in inflammatory parameters [29]. Ultrapure water could therefore be a solution to reduce dialysis related outbreaks.

### NICU and neonatal incubators

A neonatal incubator is used for premature infants to provide a thermoneutral environment. This is necessary to increase the survival chances of premature infants. Humidification of the incubator reduces the rate of evaporation and therefore reduces evaporative heat loss.

**Fig. 3:**
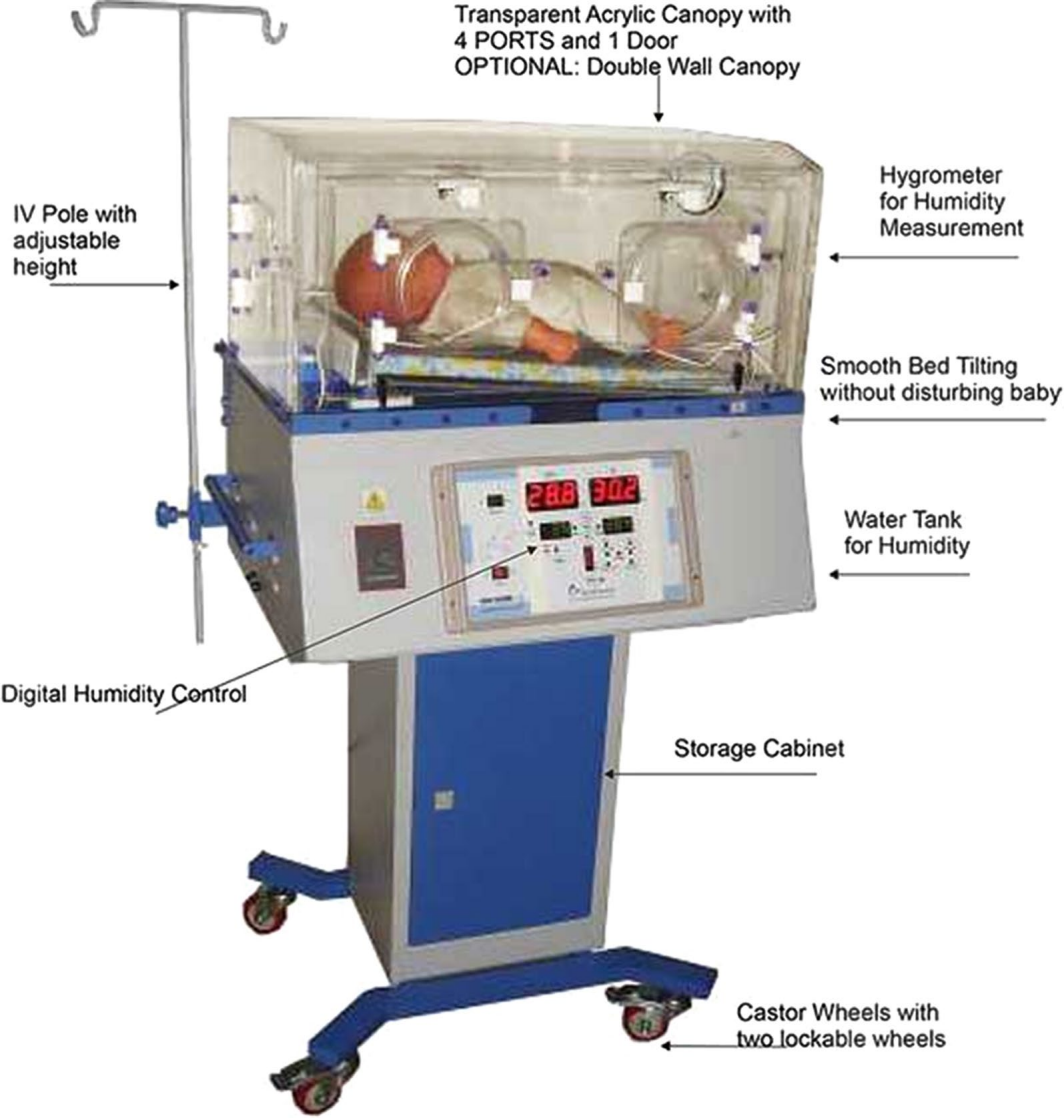
An example of a neonatal incubator [30]

The humidification chamber of the incubator contains a reservoir of water, usually distilled water, and a heating element. Evaporation occurs as the water is heated. The fresh air flow is passed through the humidification chamber so that it can be saturated with water vapour (see Fig. 3). This can either occur by allowing the fresh air flow to pass over the water, bubble through the water or come into contact with wicks dipped in the water, thereby dramatically increasing the surface area available for evaporation [31]. The warm humidified air is then blown into the incubators from hot air vents. The warm and moist habitat within an incubator is ideal for microbial growth [32, 33] (see Fig. 3). We found 7 reports that reported outbreaks related to a water reservoir of neonatal incubators (Table 4).

**Table 4:** An overview of all the neonatal incubator related outbreaks in hospitals

Study	Country	Strain	N of cases	Mortality, N (%)	Source
Mutlu. et al. [34]	Turkey	Sp*hingomonas paucimobilis*	13	1 (7.7)	Probably water that is used for humidifying and mechanical ventilators
Etienne et al. [35]	United States	*A*sp*ergillus fumigatus*	3	1 (33.3)	(Water of) humidity chamber in incubator
Yiallouros et al. [36]	Cyprus	*Legionella pneumophila*	9	3 (33.33)	Cold mist ultrasonic humidifier in the nursery
Kendrirli et al. [37]	Turkey	*Ralstonia picketti*	2	1 (50.0)	Distilled water used for humidification in the ventilator circuit got contaminated
Lee et al. [38]	Malaysia	*Burkholderia cepacia*	23	2 (8.7)	Ventilator water trap and humidifier trap
Jeong et al. [39]	Korea	*Klebsiella oxytoca*	6	Unknown	Water reservoirs of humidifiers attached to the incubators
Ebenezer et al. [31]	India	*Acinetobacter baumanni*	6	1 confirmed (16.7) 4 probable^a^ (66.7)	Oxygen humidifying chambers

^a^These infants were discharged at parental request and these were taken home terminally ill by the family

### Infection control

It is important to follow the manufacturer’s guidelines. It was seen in one study that the humidifier’s reservoirs of the incubators were filled with tap water, instead of the recommended distilled water. This resulted in an outbreak of *Pseudomonas aeruginosa*. This led to 8 bloodstream infections In neonates, of whom 2 died [40]. Therefore it is important to follow the manufacturer’s instructions. Other safety measures could include new designs where the immersion heater is placed in a way that a small amount of water is boiled just before the humidity is disbursed into the air circulation within the compartment. In this way, sterile humidity is created and offered to the neonate in a gaseous state with no airborne water droplets as vectors of microorganisms [33].

Others have noticed that the traditional humidification systems have tubing placed distal to the boiler to conduct steam from the boiler to the compartment. It is suggested that such tubing would allow re-condensation of water vapour into a liquid state and thus, acting as a reservoir for bacterial growth [33]. New designs where the tubing is placed in a different way could resolve this.

A more feasible solution includes a high-efficiency particulate air (HEPA) filtration to minimize the microbial contamination. It can also be implemented by using portable HEPA units. Sterilized incubators should be wrapped with transparent plastic sheets and stored away [35].

### Dental units and dental unit waterlines

**Fig. 4:**
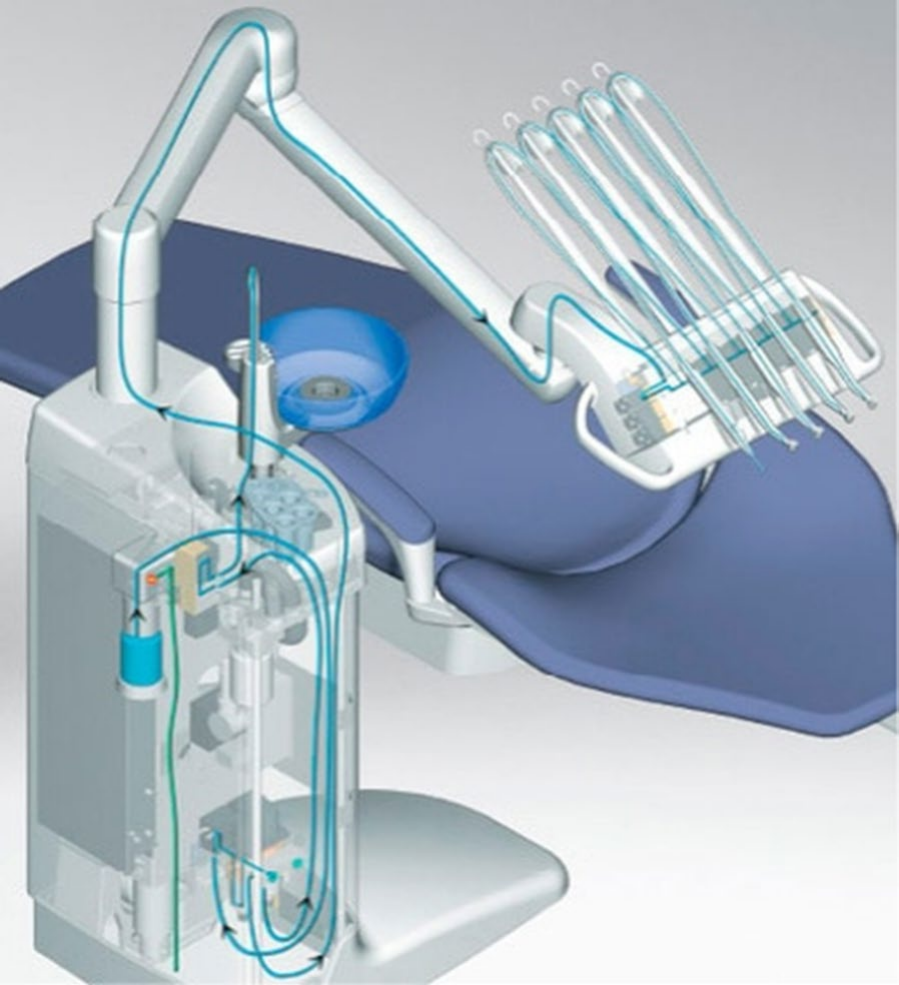
An example of a dental unit waterline [42]

A dental unit waterline (DUWL) is a complex system that delivers water to different points: water bottle tanks, glasses for patients, handpieces for high-speed drills, ultrasonic scalers, and air and water syringes. Water is used to cool dental instruments and also to irrigate tooth surfaces during dental procedures, as the heat that is generated during usage can be harmful to teeth. Water from DUWLs can also be used for oral rinsing to wash out the dental chair unit spittoon, or cuspidor, after oral rinsing (water supplied via the bowl‐rinse outlet) [41] (Fig. 4).

Generally, dental units are equipped with a dual water supply system that allows the system to be supplied with municipal water or sterile water or with both types. Type A is provided by a water tank and type B is directly connected to municipal water. Often, type A DUWLs are more contaminated than type B DUWLs [43].

DUWL contamination is often caused by the municipal water and the oral cavities of patients by aspiration of biological fluid during therapy. Thus, if dental patients are pathogen carriers, microorganisms can be transmitted by either direct contact or through aerosol sprays created by dental handpieces. Nowadays, most DUWLs contain anti-retraction valves to stop or remove any suck-back of oral contaminants [43, 44].

Biofilm in DUWLs is caused by different factors, such as water stagnation due to inactivity when patients are not treated, anti-retraction valves failure, the presence of water heaters (maintaining temperatures over 20 °C), and variations in the type of water supply (tap water, distilled water, or sterile water) [43, 44].

Additionally, the laminar flow of water that passes through a DUWL is maximal at the center of the lumen and less at the periphery, which favours the deposition and adhesion of microorganisms to the inner surface of the tube and, thus, promotes biofilm formation [43, 44].

A previous study has observed that the type of water used for a DUWL plays an important role. Sterile and distilled water is more favourable than tap water [45]. Another important factor is the setting of the DUWL. These  DUWLs are commonly used in dentistry practices where often healthy people visit the dentist. However, DWULs can also be used in clinical settings such as oral and maxillofacial surgery, oral surgery, orthodontics, paediatric dentistry and restorative dentistry. In these cases, microbial contamination could be a potential source of cross-infection and should be highly taken into account [41]. Up until now, there is limited research on outbreaks of DUWLs in clinical settings.

Studies have demonstrated that, among the microbiological contaminants of DUWLs, Pseudomonadaceae species, including *Burkholderia cepacia*, *Chryseomonas luteola*, *Pseudomonas fluorescens*, *Ralstonia pickettii,* and *Sphingomonas paucimobilis*, *Pseudomonas aeruginosa*, *Legionella pneumophila* and non-tuberculous mycobacterial species are the most highly represented among isolated and identified microorganisms [43, 44, 46, 47].

### Infection control

All dental instruments that are connected to a DUWL and are used in the patient’s mouth should contain anti-retraction valves to prevent backflow or back siphonage of fluids from the oral cavity into the DUWL [41]. According to the Dutch national guidelines all handpieces should be operated to discharge water for a minimum of 10 s after each patient. Microorganisms from a patient’s mouth can end up in the handpieces and the water pipes, ultimately causing contamination of the water in the pipes of the unit. Anti-retraction valves are not an ideal solution, as they are not able to fully retain all microorganisms. Therefore the water pipes should also be flushed for 10 s after every patient to remove these microorganisms [48]. All dental handpieces should also be cleaned, lubricated and sterilized by autoclaving after each patient use [48, 49].

In addition to this, air–water syringes should be flushed with air and water for a minimum of 10 s after each patient. An air–water syringe either consists of a disposable or reusable tip. Reusable tips are hard to clean and require cleaning and thermal disinfection. In cases when there is no adapter available for the cleaning device, the tips should be internally cleaned with an interdental brush, flushed and then sterilized in a class B autoclave [48]. Another recommendation is to not use heated water. Some DUWLs are equipped with heaters to warm the water to make it more comfortable for the patient. However, this promotes the proliferation of Legionella bacteria. Therefore, it is not recommended to equip DUWLs  with heaters unless adequate control measures are being taken [50].

There are existing nonchemical and chemical methods to decrease the presence of biofilm. Nonchemical strategies include flushing, drying and applying an antimicrobial filter. However, these methods do not appear to be effective [43].

On the other hand, flushing out the water from handpieces is useful to eliminate the stagnant water in the pipes after an inactive period. Flushing generates a pressure suitable to remove bacteria that adhere weakly to biofilm. Water pre-treatment filters or microbial filters at the ends of DUWLs may also be beneficial in treating the supply water However, these measures do not have an effect on existing biofilm [41, 43].

Chemical agents such as sodium hypochlorite or hydrogen peroxide have been demonstrated to be more effective, but need to be performed correctly to ensure the effectivity. It has been shown that a combination of nonchemical and chemical agents work synergistically [43]. After chemical treatment, the DUWLs should be flushed thoroughly with clean water [41].

It should be noted that these chemical treatment agents have not been developed by DUWL-manufacturers. They were manufactured in response to the needs of dental workers. There is a potential for incompatibility of DUWL treatment agents with components of the DWUL network and its instruments [50].

### Fluid warmer

A fluid warmer delivers normothermic fluid at routine flow rates for warming fluids such as crystalloids, colloids or blood substitutes. It is typically used to treat hypothermia. A warmer with a pump is used to circulate warmth-transferring fluid. A triple lumen tubing is used to allow blood or fluid to be warmed while traveling through the sterile center lumen while heated fluid flows through the outer lumens, enveloping the center lumen in warmth.

The water reservoir is filled with sterile water that should be changed monthly. The inside should be monthly cleaned with a 30% alcohol solution and rinsed twice with distilled water. Bleach is not recommended due to the potential damage [51].

There is not much data regarding contaminated fluid warmers. We found three articles that identified breached fluid warmers. One study observed > 100,000 colonies of multiple gram negative organisms in the water. This was likely the cause of an open port that allowed water to spill out and potentially contaminate gloved hands during use [52]. Another study reported the presence of *Pseudomonas pickettii* [53]. The third study reported that the patient experienced a transient bacteraemia, but that the isolates from the fluid warmer did not match. Exact culture results are not given [54].

### Infection control

The fluid warmer can cause leakage of the water bath solution directly into the patient via IV lines. Two cases reported that water for the circulating water bath was seen coming out of the patient end of the line. Upon further inspection, a small hole was discovered. Therefore it is necessary to check if the fluid warmer is still intact and to check the integrity of the lines of the fluid warmers before using them. The holes did not appear to be the result of mishandling or faulty installation. Unfortunately, blood leak detectors are not available in fluid warmers [53, 54].

There are a few ways to check for possible leaks. One way is to put methylene blue in the reservoir with sterile water, but this method is not confirmed by the manufacturer. Another way to check if the fluids administered contain either glucose or blood is to use Hemastix. The final method is to prime the circulating water bath and then remove the plugs covering the IV’s connections before priming the patient line. A disadvantage of this is possible contamination during priming [53].

In addition, there are water-free alternatives that would have the preference from an infection control perspective.

### Nebulizer

**Fig. 5:**
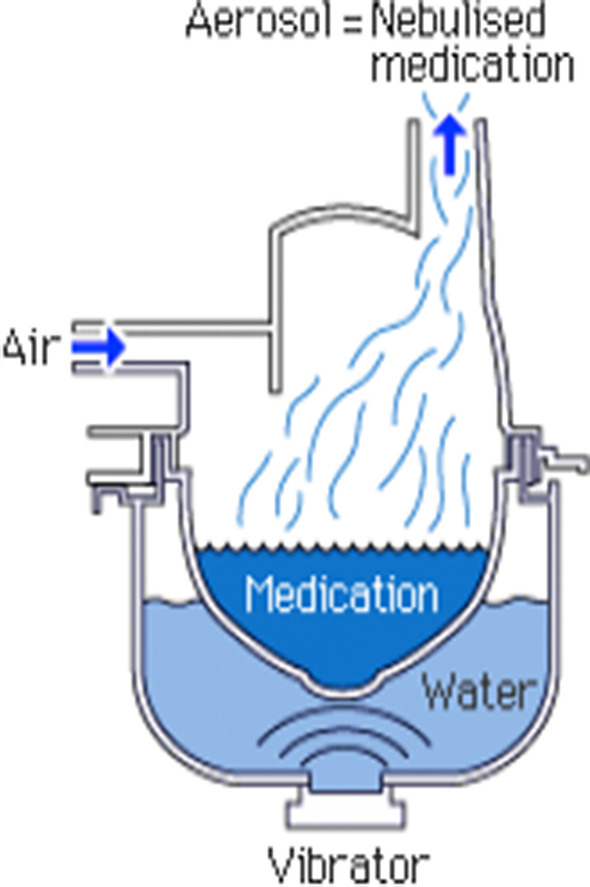
An example of a nebulizer [57]

A nebulizer is a device that turns solutions of respiratory medicine into a mist to be inhaled (see Fig. 5) They are used to administer broncho-dilating agents in acute exacerbations of COPD patients, hypertonic saline to liquify mucus in bronchiectasis, and antibiotics for chronic respiratory infections, such as tobramycine or colistin for cystic fibrosis patients with a *Pseudomonas aeruginosa* infection or liposomal amikacine for pulmonary infections with *Mycobacterium avium complex.* Nebulizers can also be used to humidify the air for tracheostomy patients. An ultra-sonic nebulizer typically has a water reservoir. Ventilation enables airflow to cross the nebulizer and to expel the aerosol droplets. Since nebulizers are hand-held devices they can also be used at home. Studies have shown that domiciliary nebulizers were often contaminated with bacteria at concentrations that could be inhaled [55, 56] (Table 5).

**Table 5:** An overview of all the nebulizer related outbreaks in hospitals

Study	Country	Strain	N of cultures, %	N of cases	Mortality, n (%)
Schloesser et al. [58]	Germany	*Acinetobacter calcoaceticus*	41 out of 90 (45.6)	7	Unknown
Schultsz et al. [59]	The Netherlands	*Methicillin-resistant Staphylococcus Aureus*	NA	17	Unknown
Craven et al. [60]	United States	Gram negative bacilli	13 out of 19 nebulizers (68.4)	NA	NA
Cobben et al. [61]	The Netherlands	*Pseudomonas aeruginosa*	4 out of 22 nebulizers (18.2)	21	4 (19.0)
Takigawa et al. and Yamagishi et al. [62, 63]	Japan	*Burkholderia cepacia*	NA	37	4 (11.1)

We found 5 reports of nebulizer-associated outbreaks in a hospital setting. One study found an outbreak of *Acinetobacter calcoaceticus* and speculated that central venous catheters, the oral cavity or the umbilicus served as portals of entry in the blood stream [58].

### Infection control

In the study of Schultsz et al. [59] a Dutch hospital had an outbreak of MRSA and found that the nebulizer was a potential source. The outbreak was likely due to incorrect cleaning of the nebulizer as maintenance procedure indicated that the tubing, pot and sterile water of the nebulizer was changed twice a week, but the dust filter was not washed weekly, despite being advised in the maintenance protocol. Furthermore, no cases of MRSA were found after vacuuming, washing, disinfecting, and installing new dust filters and cleaning those filters on a weekly basis.

One study found that contamination rates dropped from 68 to 20% when nebulizers were cleaned after each treatment [60]. Therefore, it is important to highlight the necessity of proper handling and cleaning of the nebulizer. The nebulizer should only be filled when it needs to be used and should be cleaned every 24 h. Water or sodium chloride 0.9% that is used to dissolve medication should be sterile and preferably from a single container that is stored in a fridge. Containers should not be used if they have already been opened for more than 24 h. It is also important to note that when filling the nebulizer with medication, the dropper tip should not touch the nebulizer reservoir. One study observed that therapists frequently tapped the dropper tip against the nebulizer reservoir to free the last drop of solution and then inserted the dropper back into the bottle of medication [64]. It is recommended to use removable parts, so the devices can be disassembled and should be flushed with lukewarm water and disinfected every day with 70% ethanol and sterilized by autoclaving weekly, after which they should be air dried. When using disposable tubes, they should be replaced every day. Nebulizers should not be shared to prevent cross-infection [62, 65] (Fig. 5).

### Water traps

**Fig. 6:**
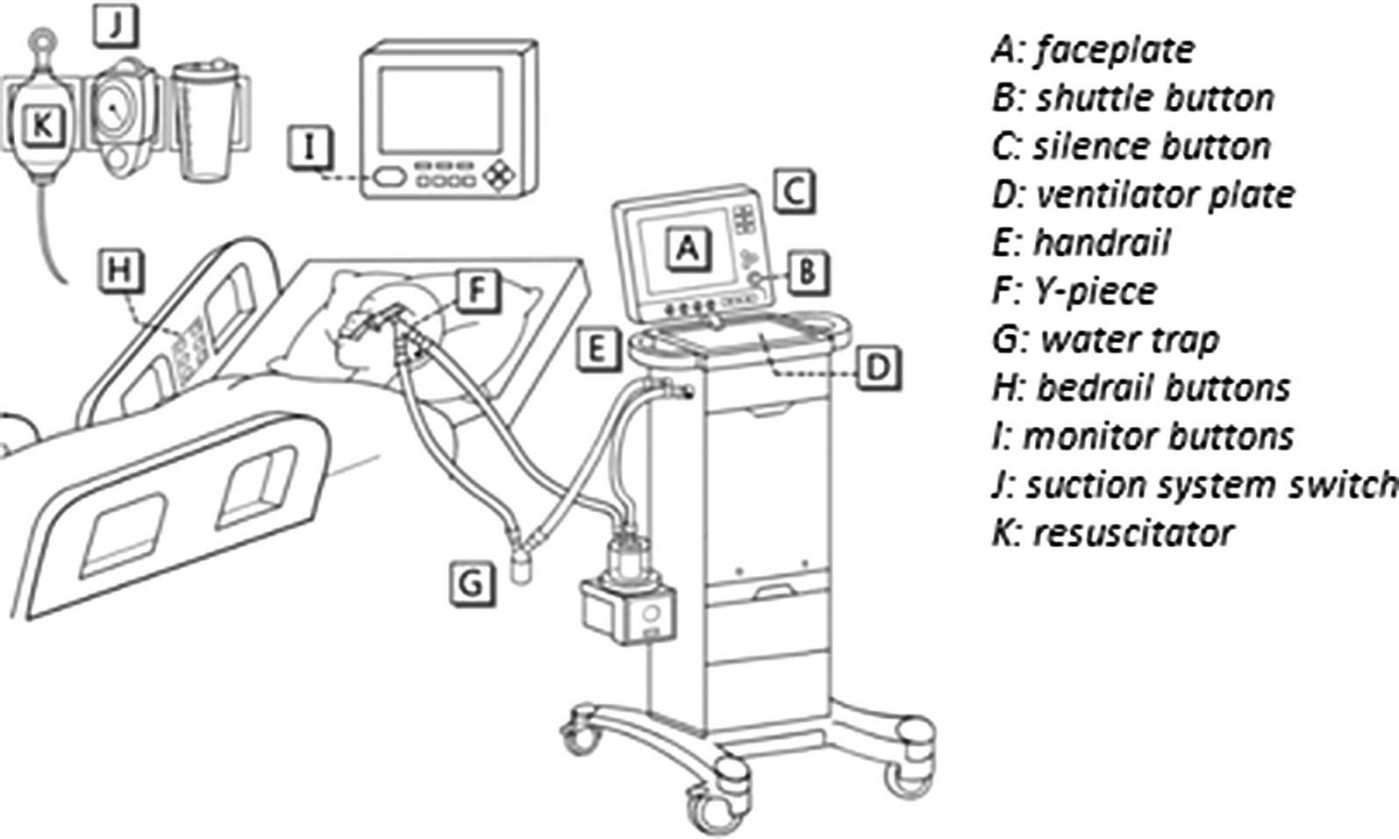
An example of a ventilator circuit system set-up. The water trap is indicated by G [66]

Most mechanical ventilators make use of water traps. Water traps collect condensation in breathing circuits to prevent it from damaging the ventilator or flowing backing to the patient and are also used during gas measurements to prevent water from entering the gas analyser (see Fig. 6). They are strategically placed in the circuit to catch condensate. Water traps can be either disposable or reusable.

Our literature review identified 4 reports of outbreaks associated with water traps. Studies have shown that the water condensate in the traps is a reservoir for potential outbreaks if not handled properly [69] (Table 6).

**Table 6:** An overview of all the water traps related outbreaks in hospitals

Study	Country	Strain	N of cultures, (%)	N of cases	Mortality, N (%)	Source
Sui et al. [66]	Taiwan	1. *Pseudomonas aeruginosa* 2. *Staphylcoccus aureus*	N = 15 water traps 1. 2 (13.3) 2. 7 (46.7)	NA	NA	Water trap
Gorman et al. [67]	Scotland	*Klebsiella pneumoniae*	NA	6	2 (33.33)	Ventilator expiratory water trap
Lee [38]	Malaysia	*Burkholdera cepacia*	NA	23	2 (8.7)	Ventilator water traps and ventilator humidifier trap
Kaul et al. [68]	Canada	*Acinetobacterbaumanii*	Outbreak investigation on only 5 out of 7 ICUs: 1 out of 95 traps (1.1)	NA	NA	Water traps

### Infection control

According to the Dutch WIP (Working party on Infection Prevention) guidelines water traps should be periodically drained and discarded. They should be replaced after a week, at the same time as the tubes of the ventilation circuit, when the water traps are full or when they are evidently polluted, or when the patient is discharged [70, 71]. However, tubes connecting to and from the water traps should be aired every day and they should be reconnected diligently [72]. When using reusable water traps sterilization or thermal disinfection should be applied [71]. The device should be positioned below the bed level to prevent drainage towards the patient. The ventilator tubing should always be drained before repositioning the patients [73, 74].

The condensate should be considered contaminated waste and should therefore be minimally handled and disposed of through the standard hospital waste stream. When opening or breaking the circuit to drain the condensate, there is a potential for caregiver exposure to condensate during the ventilator or water trap disconnection or disposal. Therefore it is important to apply standard hand hygiene [70]. Additional consequences related to breaking the circuit, but not solely on the water trap include:

1. Potential for contamination of the interior of the circuit
2. Potential for cross-contamination of other patients
3. Loss of PEEP and/or de-recruitment of the lung [69]

During these circuit disconnects, ventilators may generate a high flow through the patient circuit that may aerosolize contaminated condensate. Therefore, it is recommended to remove condensate from ventilator circuits and keep the ventilator circuit closed while doing so to minimize contamination [69]. One study has observed that while the traps were disconnected, the condensate was dripping on the floor and no proper cleaning measures were taken. In case of spillage, the condensate should have been absorbed on to paper towel and discarded as clinical waste. Then the floor should be washed with hot water and detergent [67].

Disposing the condensate is also essential. Several studies have observed that the condensate was emptied into the bedside sink, garbage bin, foil dishes beside the bedside [67]. This can still cause aerosolization and thus, inhalation by the patient. The condensate should be disposed immediately away from the patient.

### Water bath

Water baths are often used to thaw cryoprecipitate. Cryoprecipitate is made from fresh-frozen plasma and used to treat bleeding disorders and to manage large-volume bleeding, such as operating rooms, obstetric practice, and emergency departments. Current FDA standards require that cryoprecipitate must be transfused within 6 h after thawing. Extended storage of thawed cryoprecipitate at room temperature may increase the risk of bacterial contamination [75, 76]. The processes used to thaw and store cryoprecipitate can also determine whether contamination occurs. We identified two case reports of *Pseudomonas* septicaemia after plasma transfusion [76] during the studied time period (see Table 7).

**Table 7:** An overview of all the water baths related outbreaks in hospitals

Study	Country	Strain	N of cultures (%)	N of cases	Mortality, N (%)	Source
Muyldermans et al. [77]	Belgium	*Pseudomonas aeruginosa*	NA	4	3 (75.0)	Water bath to warm fresh frozen plasma and human albumin
Casewell et al. [78]	England	*Pseudomonas aeruginosa*	2 out of 9 experiments (22.0)	1	1 (100.0)	Water bath’s water
Yuen et al. [79]	Hong Kong	1. *Acinetobacter anitratus* 2. *Pseudomonas putida* 3. *Bacilus subtilis* 4. *Pseudomonas paucimobilis* 5. *Tricho*sp*oron beigelii* 6. *Corynebacterium* sp*.* 7. *Candida tropicalis*	All 3 water baths (100.0)	5 patients with Candida tropicalis	3 (60.0)	Water bath’s water likely contaminated the nurses’fingers. In addition, removable metal grid was not cleaned at the bottom, because personnel assumed it was fixed to the base

### Infection control

There are no clear guidelines on how to disinfect the water baths. They should be emptied and cleaned at the end of each day and stored dry overnight. However, from an infection control perspective these water baths are not preferred in the patient environment due to contamination risks. In the study of Casewell et al. [78] it was reported that there was doubt whether this cleaning process was performed during busy periods. After the fatal case, the policy was revised and no incidents occurred after the renewed policy. All surfaces and probes should be cleaned with hot water and detergent using a sterile Magill brush after usage. Afterwards, when the surfaces are thoroughly dry, they should be sprayed with 0.5% chlorhexidine in 70% spirit. Immediately before use, all surfaces are sprayed again with alcohol chlorhexidine and the bath should be filled with sterile distilled water. Whilst in use, the water baths should be changed routinely every 4 h.

Additionally, bags of cryoprecipitate should be handled with care as there are brittle and may be fractured whilst being thawed. They should not be removed from the cardboard carton until thawed and they should not be massaged to accelerate thawing. Blood product should also be double-bagged in a sterile outer bag which can only be opened after thawing [78]. Wet-warming dialysate is not recommended. Dialysate should be warmed by dry-warming methods such as electric blankets or warming cabinets [79].

There have also been suggestions to replace all water baths for heating of blood components by dry systems such as dry heating incubators to reduce the risk of microbial contamination [77, 80] (see Table 8).

**Table 8:** An overview of all the water containing devices in hospitals

Medical equipment	N of articles	Risks	Transmission route	Patient population	Prevention
Heater-cooler unit	> 10 due to global outbreak	Ventilators	Airborne through the airflow of the HCU into the operating room	Cardiopulmonary bypass surgery patients	Use different kind of HCU Place HCU as far away from the patient
Hemodialysis equipment	11	Water purity Improper functioning → backflow	Through healthcare workers. Gloves as fomite Direct contact through backflow of the dialysis line	Dialysis patients	Water with microbial contamination of < 0.1 CFU/mL and endotoxin contamination of < 0.03 IU/mLExtra filter
Neonatal incubators	7	Humidity regulation	Aerosolization into incubator Through healthcare workers. Gloves as fomite	Premature neonates	Extra HEPA filter Possible new designs
Dental units	> 10	Water stagnation Aerosol droplets	Direct contact with contaminated water due to biofilm that is formed during water stagnation Aerosol droplets due to the use of dental devices	Dental patients	Nonchemical and chemical agents to prevent and remove biofilm
Fluid warmers	3	Integrity of the membrane	Direct contact with the patient’s blood through a hole in the fluid warmer	Patients suffering from hypothermia, often in high risk departments (ICU, surgery, A&E)	Proper handling prior to using to check the integrity of the tubes
Nebulizers	5	Aerosol droplets	Through healthcare workers. Gloves as fomite Aerosolization into room air Direct airway inoculation through connected ventilation system	patients with COPD, pneumonia or tracheostomy patients	Proper cleaning and handling Avoid multiple users
Water traps	4	Water condensate	Through healthcare workers. Gloves as fomite Aerosolization of water condensate	Patients who receive respiratory care through a ventilator: surgery and at ICU	Proper cleaning and disposal of the condensate
Water baths	3	Water in water baths Integrity of the bag	Hands of healthcare workers. Gloves as fomite Direct contact due to fracture in the bag	Patients who need cryoprecipitate transfusion	Proper cleaning of the water bath Handle the cryoprecipitate with care Replace the water baths

## Results: summary

### Discussion

Water containing medical equipment can be a source of transmission and cause an outbreak. This risk is likely to be underestimated by healthcare workers, in particular for devices without clear cleaning and disinfection guidelines.

In most cases an outbreak occurred due to improper cleaning of the medical device or using the wrong kind of water. As seen in the article of Weng et al. [40] the outbreak at the NICU was clearly caused due to the tap water. The article of Susantitaphong et al. [29] highlights the beneficial effects of using ultrapure dialysate in comparison to standard dialysate. Up until now, clear guidelines are still lacking for many medical devices. Methods still vary widely and there is no consensus on how to properly clean the device. In addition to routine basic infection control measures as well as the monitoring of the equipment or systems by collecting routine samples to determine the microbiological status, this review provides recommendation for reducing the risk of transmission from water-containing medical equipment.

### How can we do better?

#### Role of the manufacturer

The easiest way to prevent outbreaks associated to water containing medical equipment is to eliminate the use of water. Manufacturers should consider developing devices that do not use water. Manufacturers should develop devices that come with clear and feasible cleaning and disinfection instructions. It is important that manufacturers also consider whether these instructions also align with the approved cleaning and disinfections products of the country where the device will be used. Ideally, medical equipment is designed in way that facilitates easy cleaning and disinfection, and infection control experts as well as end users are involved in the design process.

There should be clear manufacturer’s guidelines and regular revisions. The HCUs serve a good example for how manufacturers should react. When the outbreak occurred, the manufacturer revised and intensified their guidelines multiple times. This made it easier for healthcare staff, since they could implement these measures without losing time to figure out appropriate safety and cleaning measures themselves. Some safety measures require a lot of time, money and effort, such as placing the HCU outside the operating room or implementing a new incubator design. These measures were all proven to be more favourable than merely an additional filter, as shown for the HCUs. However, without proper guidelines it is less likely that hospitals will take a gamble in additional safety measures and will choose these ‘difficult’ solutions as they might be afraid that their efforts will go to waste.

Additionally, clear guidelines provide an overview of the various safety measures that are proven to be compatible with the device. Incompatibility was shown to be an obstacle when treating DWULs with chemical agents and could lead to a suboptimal effectiveness [50].

#### Role of the hospitals

The purchase of new medical equipment ideally goes in consultation of the Infection Prevention staff. Even though guidelines or manufacturer’s instructions exist for every device, not everyone followed them through, as was the case in most outbreaks related to the nebulizers.

Most outbreaks occurred due to improper cleaning of the device, such as in the article of Weng et al. [40]. Despite manufacturer instructions, the healthcare personnel still deviated from the cleaning instructions, which ultimately lead to an outbreak. Cleaning instruction should be followed thoroughly, especially for devices that are exposed to multiple users. Re-using and exposing devices to multiple users was not recommended for dialysis equipment and nebulizers. For the water bath, there was even a case where cleaning procedures were neglected due to busy periods [78]. This shows that cleaning procedures should be more prioritized.

Therefore, healthcare personnel should be adequately trained on basic infection control, such as using gloves and disinfecting. Many outbreaks that occurred were caused by improper usage of gloves by healthcare staff, acting as a fomite [81]. This is especially important to prevent cross-infection when healthcare workers see many patients per day such as people working at the NICU or dental workers.

An example to highlight the importance of this are the DWULS, which are often treated with chemical agents. Improper handling could lead to adverse effects for both the healthcare staff and the patients [41].

Since all the mentioned devices contain a water reservoir, it should also be important to train healthcare workers how to dispose the content when cleaning the device. The consequence of incorrect disposal in water traps show that it can still cause aerosolization and thus, inhalation of contaminated water. It is important to note that when working with these water containing devices that should not come in contact with the patient and the healthcare workers and their environment, users should always check the equipment before usage. Factors that could influence the integrity of a membrane or tube, such as a hole, can cause direct contact with possible contaminated fluid. This could be seen in the fluid warmers and hemodialysis equipment, where it was only detected afterwards that the device was malfunctioning due to a breach in the tube [53]. Inspection before usage is an easy safety measure, but often neglected due to the lack of time.

Furthermore, this thesis has shown that there is a need for clearer guidelines and training of medical staff to prevent possible outbreaks in the future.

### Limitations

Due to the limited research on this topic, no literature could be found on the blanketrol, the scalp cooling used for chemotherapy and thermic stimulators. These medical devices could still be prone to microbial growth and contamination. This is an important research gap and it should be covered in future research to guarantee the safety of a device.

Most literature regarding the DWULs were in a dental setting where mainly healthy patients visit. It is still unknown what the risks are for DWULs in a more clinical setting. This can also be seen in the nebulizers where more literature was available on domicile usage than in a healthcare setting. It is likely that the risks are higher in clinical settings as certain devices are used by multiple patients and these patients are often more immunocompromised or exposed to surgery. For future research, it is recommended to look more at clinical settings with more immunocompromised patients to fully understand the potential risks.

Another limitation and research gap is that not much is known about outbreaks related to water containing hospital equipment in higher income countries. The literature found in this thesis mainly consists of data in lower and middle income countries, in particular for hemodialysis and the neonatal incubators. It is unknown whether the prevalence of these outbreaks in higher income countries are in fact lower or if they are not described and documented. This also contributes to publication bias. Not all healthcare-associated outbreaks are being published. Hence, there could be more HAIs that were not covered due to the lack of documentation. Therefore more research in these settings are favourable to assess whether high income countries are facing similar risks.

Many more reservoirs for microbial growth could be possible but they remain unknown, because they are not described in outbreaks or case reports. Reservoir detection requires extensive investigation and long-time experience. Therefore it is difficult to exactly pinpoint the common reservoir of an infection outbreak, because colonized patients and healthcare workers can become a secondary source of infection and there are multiple transmission routes possible [3].

## Conclusion

In conclusion, many water containing medical equipment in hospitals are potential reservoirs for microbial growth and contamination. Proper handling and cleaning can help to reduce the microbial burden and reduce the biofilm that can potentially cause an outbreak. Clear manufacturer guidelines and instructions are needed to help medical staff in order to achieve this. Medical staff should be trained as well to raise awareness of the importance of proper handling and cleaning of such medical equipment.

Furthermore, manufacturers should be stimulated to develop devices without fluid reservoirs for microbial growth. Hence they should always search for water free alternatives. If fluid reservoirs are necessary, the manufacturer must develop the device in a way that the risk of biofilm formation is minimal and cleaning and disinfection procedures are feasible in the practical setting of an hospital/medical organisation. Before purchase of medical device by an organisation, the clinical physics department and the infection prevention department must be involved.

More research is needed on the water containing hospital equipment that was not covered in this thesis due to lack of literature. More research in high income countries will also help to assess the actual risks when translating the research into practice. This could possibly also reveal more potential reservoirs for microbial growth.

## Data Availability

Not applicable.

## Abbreviations

CVC: Central venous catheter
DUWL: Dental Unit Waterline
ECMO: Extracorporeal membrane oxygenation
HAI: Healthcare-associated infection
HCU: Heater-cooler unit
WHO: Waste Handling Option
